# Methicillin Resistant *Staphylococcus aureus* Transmission in a Ghanaian Burn Unit: The Importance of Active Surveillance in Resource-Limited Settings

**DOI:** 10.3389/fmicb.2017.01906

**Published:** 2017-10-06

**Authors:** Nana Ama Amissah, Andrew H. Buultjens, Anthony Ablordey, Lieke van Dam, Ampomah Opoku-Ware, Sarah L. Baines, Dieter Bulach, Caitlin S. Tetteh, Isaac Prah, Tjip S. van der Werf, Alexander W. Friedrich, Torsten Seemann, Jan Maarten van Dijl, Ymkje Stienstra, Timothy P. Stinear, John W. Rossen

**Affiliations:** ^1^Department of Internal Medicine/Infectious Diseases, University of Groningen, University Medical Center Groningen, Groningen, Netherlands; ^2^Department of Bacteriology, Noguchi Memorial Institute for Medical Research, University of Ghana, Accra, Ghana; ^3^Department of Microbiology and Immunology, The Doherty Institute for Infection and Immunity, University of Melbourne, Melbourne, VIC, Australia; ^4^Department of Medical Microbiology, University of Groningen, University Medical Center Groningen, Groningen, Netherlands; ^5^Burns Unit, Reconstructive Plastic Surgery and Burns Unit, Korle Bu Teaching Hospital, Accra, Ghana; ^6^Victorian Bioinformatics Consortium, Monash University, Clayton, VIC, Australia

**Keywords:** *Staphylococcus aureus*, burn patients, transmission events, comparative genomics, healthcare workers

## Abstract

**Objectives:** *Staphylococcus aureus* infections in burn patients can lead to serious complications and death. The frequency of *S. aureus* infection is high in low- and middle-income countries presumably due to limited resources, misuse of antibiotics and poor infection control. The objective of the present study was to apply population genomics to precisely define, for the first time, the transmission of antibiotic resistant *S. aureus* in a resource-limited setting in sub-Saharan Africa.

**Methods:** *Staphylococcus aureus* surveillance was performed amongst burn patients and healthcare workers during a 7-months survey within the burn unit of the Korle Bu Teaching Hospital in Ghana.

**Results:** Sixty-six *S. aureus* isolates (59 colonizing and 7 clinical) were obtained from 31 patients and 10 healthcare workers. Twenty-one of these isolates were ST250-IV methicillin-resistant *S. aureus* (MRSA). Notably, 25 (81%) of the 31 patients carried or were infected with *S. aureus* within 24 h of admission. Genome comparisons revealed six distinct *S. aureus* clones circulating in the burn unit, and demonstrated multiple transmission events between patients and healthcare workers. Further, the collected *S. aureus* isolates exhibited a wide range of genotypic resistances to antibiotics, including trimethoprim (21%), aminoglycosides (33%), oxacillin (33%), chloramphenicol (50%), tetracycline (59%) and fluoroquinolones (100%).

**Conclusion:** Population genomics uncovered multiple transmission events of *S. aureus*, especially MRSA, within the investigated burn unit. Our findings highlight lapses in infection control and prevention, and underscore the great importance of active surveillance to protect burn victims against multi-drug resistant pathogens in resource-limited settings.

## Introduction

In sub-Saharan Africa, burn injuries are common and, compared to high-income countries, mortality is increased even after correction for the Total Body Surface Affected (TBSA). This is reflected by the size of burn that causes a 50% mortality rate [the lethal dose of burn injury where 50% of the burn patients die (LD50)]. Advances in burn care have improved the percentage of TBSA (77–99%) at which LD50 occurs in more affluent industrialized countries (Branski et al., 2009; Khashaba et al., 2012; Kraft et al., 2012; Ellison, 2013; Jeschke et al., 2016) compared to 19–39% for burn injury victims in sub-Saharan Africa (Tyson et al., 2013; Amissah et al., 2017). In particular, nosocomial infections pose a significant challenge to the care for burn patients in developing countries. As shown in a recent study in Ghana, the overuse and general misuse of antibiotics combined with lapses in infection control largely contribute to the poor clinical outcomes in burn patients (Amissah et al., 2017). The situation is exacerbated by resource limitations, which make it difficult to maintain hospital hygiene measures and to rapidly detect and treat infections.

Burn wounds are frequently colonized by microorganisms, such as *Staphylococcus aureus*, *Pseudomonas aeruginosa*, *Acinetobacter baumannii*, *Candida* spp., *Aspergillus* spp., *and Fusarium* spp. (Branski et al., 2009; Devrim et al., 2017). This is exemplified by studies where between 66 and 76% of patients’ burn wounds were found to be colonized by *S. aureus* within 2–7 days of admission in the hospital (Alaghehbandan et al., 2012; Taneja et al., 2013). Colonization with *S. aureus* was associated with increased TBSA, delayed wound healing, and prolonged hospitalization (Manson et al., 1992; Reardon et al., 1998; Kooistra-Smid et al., 2004). In this respect, the high level of antibiotic resistance in *S. aureus* is worrisome. Methicillin resistant *S. aureus* (MRSA) bloodstream infections are known for excess mortality and increased length of hospital stay (de Kraker et al., 2011a,b). Unfortunately, there is only limited data available for MRSA carriage and infections in Africa. Of note, MRSA was the third most common organism found in blood cultures in a South African intensive care burn unit, where 17% of the patients with MRSA-positive blood cultures died (Bahemia et al., 2015). We have recently shown that in a tertiary care hospital in Ghana, 19% of the investigated burn patients carried or were infected with MRSA. The MRSA isolates of these patients belonged to *spa*-type t928 and multi-locus sequence type (MLST) 250. Of note MRSA isolates with the same *spa* and MLST types were identified in 3% of the HCWs taking care of the burn patients (Amissah et al., 2017). The MRSA isolates from patients and HCWs displayed identical antibiotic resistance patterns suggesting possible transmission events. Other studies in Africa reported nasal MRSA carriage rates of 13–18% among HCWs (Akoua Koffi et al., 2004; Shibabaw et al., 2014; De Boeck et al., 2015), and possible MRSA transmission events between patients and HCWs are known to occur (Ben Ayed et al., 2010; Conceição et al., 2014b). Importantly, bacterial typing methods, such as *spa*-typing and MLST, have a relatively low discriminatory power. The results obtained with these methods are, therefore, only indicative for nosocomial transmission events. Hard conclusions concerning MRSA transmission and hospital outbreaks can only be drawn from whole-genome sequencing (WGS). Therefore, the present study was aimed at comparing the previously collected MRSA ST250 isolates by WGS to pinpoint possible lines of transmission. Our results show that transmission had indeed occurred, and they highlight the importance of routine surveillance of MRSA in hospitals in resource-limited settings.

## Materials and Methods

A 7-month prospective survey of *S. aureus* transmission events was conducted at the burns unit of the Reconstructive Plastic Surgery and Burn Centre of Korle Bu Teaching Hospital, Ghana, from November 2014 to May 2015. The burn unit has a 21-bed capacity for the management of patients with second and third degree burn injuries. During this period the burn unit received burn cases from the intensive care unit (ICU) of the hospital and 21 other hospitals in the country. Rigorous infection control measures were not implemented in the burn unit at the time of the study. Patient information, including age, gender, type of injury, length of stay, outcome (death or discharge), percentage of TBSA and use of antibiotics, was obtained from patient files. Patient data from the referral centers prior to admission to the burn unit was unavailable. Blood cultures, nose and wound swabs were collected from the patients upon admission and biweekly during change of wound dressing as previously described (Amissah et al., 2017). Nasal swabs were collected from HCWs (doctors, nurses, and cleaners) once per 2 months during the study period.

### Ethics Statement

The ethical committee of the Noguchi Memorial Institute for Medical Research (NMIMR) (FEDERAL WIDE ASSURANCE FWA 00001824) approved the use of clinical samples and data from patients and HCWs for this investigation. All samples were collected upon written informed consent or assent from all participants aged ≥12 years, and consent from a parent, caretaker, or legal representative of any child participant below the age of 18 years.

### Nosocomial Transmission Events of *S. aureus*

Transmission events in the burn unit were defined as transfer of *S. aureus* with the same MLST type (derived from genome sequence data) from a colonized or infected HCW/patient to another HCW/patient previously negative or positive for another MLST with a core SNP difference of ≤40 (Golubchik et al., 2013; Price et al., 2014) and an overlap in hospital stay (burn unit). Phylogenetic analyses of sub-populations (based on sequence types) using the BAPS (Bayesian Analysis of Population Structure) were used to infer the directions of transmission events.

### Microbiological and Whole-Genome Sequencing Analysis

A total of 66 *S. aureus* longitudinally collected isolates from 31 burn patients and 10 HCWs (Amissah et al., 2017) were subcultured on 5% sheep blood agar and incubated at 37°C overnight. Genomic DNA was extracted from *S. aureus* using the Ultraclean microbial DNA isolation kit (MO BIO laboratories, Inc., Carlsbad, CA, United States) according to the manufacturers’ instructions. DNA libraries were prepared using the Nextera XT v2 kit (Illumina, San Diego, CA, United States), and sequenced on the Illumina platform using 2 × 250-bp chemistry. Sequence reads were submitted to the National Center for Biotechnology Information GenBank under the Bioproject ID PRJNA295807. The raw reads were assembled into contigs using spades 3.9.0 (Nurk et al., 2013) and mapped to the fully assembled chromosome of *S. aureus* COL (ST250) (chromosome: CP000046) using snippy v3.2^1^. MLST and antibiotic resistance detection were undertaken with mlst v2.8^2^ and abricate v0.5-dev^3^, respectively. Maximum likelihood Phylogenetic trees were generated using an alignment of core genome SNPs with FastTree v2 (Price et al., 2010) under the GTR model. Trees were visualized using FigTree v1.4.2^4^.

### Phylogenetic Analyses of Genetic Populations

Bayesian Analysis of Population Structure (BAPS 6) was used to cluster (a prior of 10 depth levels and a maximum of 20 clusters were specified) the DNA sequence data into genetic populations (Cheng et al., 2013). To increase the resolution, BAPS groups were further analyzed with population-specific *S. aureus* reference strains, to maximize the size of the core genome and increase the potential to detect more variants. The reference genomes of *S. aureus* COL (ST250) (Accession no. CP000046), *S. aureus* N315 (ST5) (Accession no. BA000018.3) and *S. aureus* CC45 (ST45) (Accession no. CP006044.1) were used for BAPS populations 2, 4, and 6, respectively. For BAPS populations 1, 3, and 5, genetically close reference genomes were publicly unavailable and we, therefore, selected the best *de novo* assemblies (i.e., assemblies with the greatest N50 score) within each population to serve as references. Sequence types within BAPS populations suspected of being linked to nosocomial transmission events were further analyzed.

## Results

### Whole Genome Sequence Analysis

**FIGURE 1:**
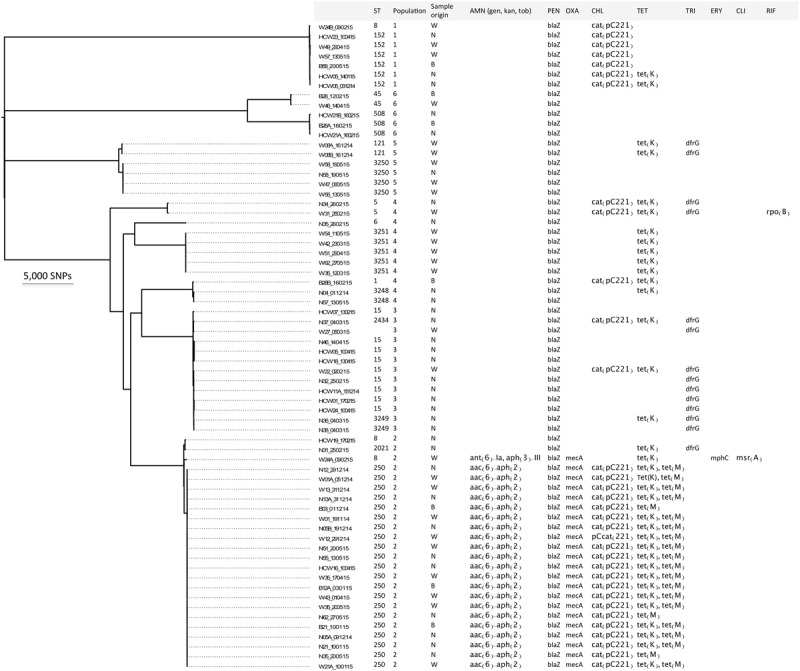
Distinct *S. aureus* populations circulating in the burn unit of Korle Bu Teaching Hospital. Maximum likelihood phylogeny based on core genome SNP analyses of the 66 *S. aureus* isolates compared to the reference strain *S. aureus* COL. The first letter(s) in the strain code represents the type of sample from patients and/or HCWs: (B) blood; (N) nose swab; (W) wound swab, and (HCW) nose swab. The following number refers to the isolate ID from patient or HCW. The third letter, (A) or (B), indicates whether more than one isolate was obtained per patient or HCW. The number after the hyphen indicates the date of sample collection. Furthermore, the figure shows the sequence type (ST), the BAPS cluster number (1 to 6), the origin of the isolate, and genotypic antibiotic resistances. AMN, aminoglycoside; gen, gentamicin; kan, kanamycin; tob, tobramycin; PEN, penicillin; OXA, oxacillin; CHL, chloramphenicol; TET, tetracycline; TRI, trimethoprim; ERY, erythromycin; CLI, clindamycin; RIF, rifampicin.

Sixty-six *S. aureus* isolates were obtained from multiple screens of 31 burn patients and 10 HCWs as previously described (Amissah et al., 2017). The first swabs from patients were collected within 24 h of admission. At this time point, 25 (81%) of the 31 patients carried or were infected with *S. aureus*, 6 (19%) of which were shown to carry MRSA. Eight patients acquired *S. aureus* in the burn unit, and in five of these cases this concerned MRSA. Upon WGS of the 66 isolates, MLST analysis identified 16 STs, including four novel types (ST3248, ST3249, ST3250, and ST3251) and one untypeable isolate (**Figure 1**). The predominant ST was ST250 MRSA. Further, these analyses revealed that one patient (i.e., patient 35) carried a ST6 *S. aureus* strain on admission and, subsequently, acquired two different *S. aureus* genotypes (ST250 and ST3251) during treatment in the burn unit.

Alignment of sequence reads for the 66 isolates against reference *S. aureus* COL defined a 2.2 Mbp core genome (79.6%) and identified 102,904 core SNPs, which were used to generate a phylogenomic tree (**Figure 1**). Interestingly *S. aureus* isolates with ST8, ST15, ST152, ST250 and ST508 were obtained from both patients and HCWs. *S. aureus* isolates with ST5, ST45, ST3248, ST3250 and ST3251 were obtained from different patients. This was indicative of transmission events between HCWs and patients, and between patients.

### Detection of Nosocomial Transmission Events within Discrete *S. aureus* Populations

A Bayesian analysis of the population structure with BAPS identified six distinct populations among the diverse collection of 66 *S. aureus* isolates (**Figure 1**). Four of these six populations (i.e., populations 1, 2, 3, and 6) comprised isolates from both HCWs and patients. The six identified populations were further analyzed using genomes from specific clonal complexes as reference strains to maximize the ability to detect strain differences. Sequence types were identified within each population and transmission events within ST clones were further investigated. In what follows, we address possible nosocomial transmission events that were revealed in the *S. aureus* populations.

**FIGURE 2:**
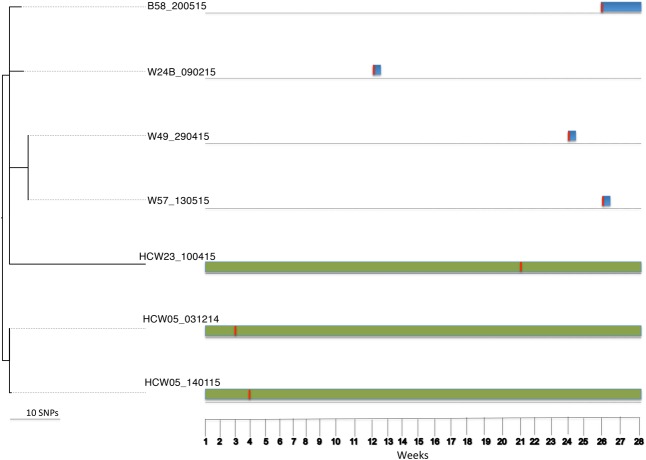
Tracing transmission events amongst burn patients and HCWs in BAPS cluster population 1 (*S. aureus* ST152). Blue and green horizontal bars represent the length of stay (in weeks) of burn patients and HCWs, respectively, in the burn unit. The red vertical lines represent the time point at which patients or HCWs tested positive for ST152.

For BAPS population 1, sequence comparison (reference isolate W57_130515) among seven isolates that belonged to ST152 identified 429 core SNPs (among a core genome of 2.7 Mbp). Within this population were isolates cultured from the nares of two HCWs, wounds of three patients and blood of one other patient. Interestingly, all four patients (i.e., patients 24, 49, 57, and 58) tested positive within 24 h of admission (first screening) (**Figure 2**). Two patients were referred from the Korle Bu Teaching Hospital’s ICU (i.e., patients 49 and 57) and two from separate hospitals (i.e., patients 24 and 58). Here, it is likely all four patients acquired the ST152 *S. aureus* strain from their referral healthcare centers prior to admission to the burn unit. The two HCWs acquired the ST152 strain at one point in time during the study period (**Figure 2**). This implies that upon introduction into the burn unit, the *S. aureus* ST152 strain was indirectly transmitted to HCWs, either through contaminated surfaces and fomites, or through other unidentified individuals.

**FIGURE 3:**
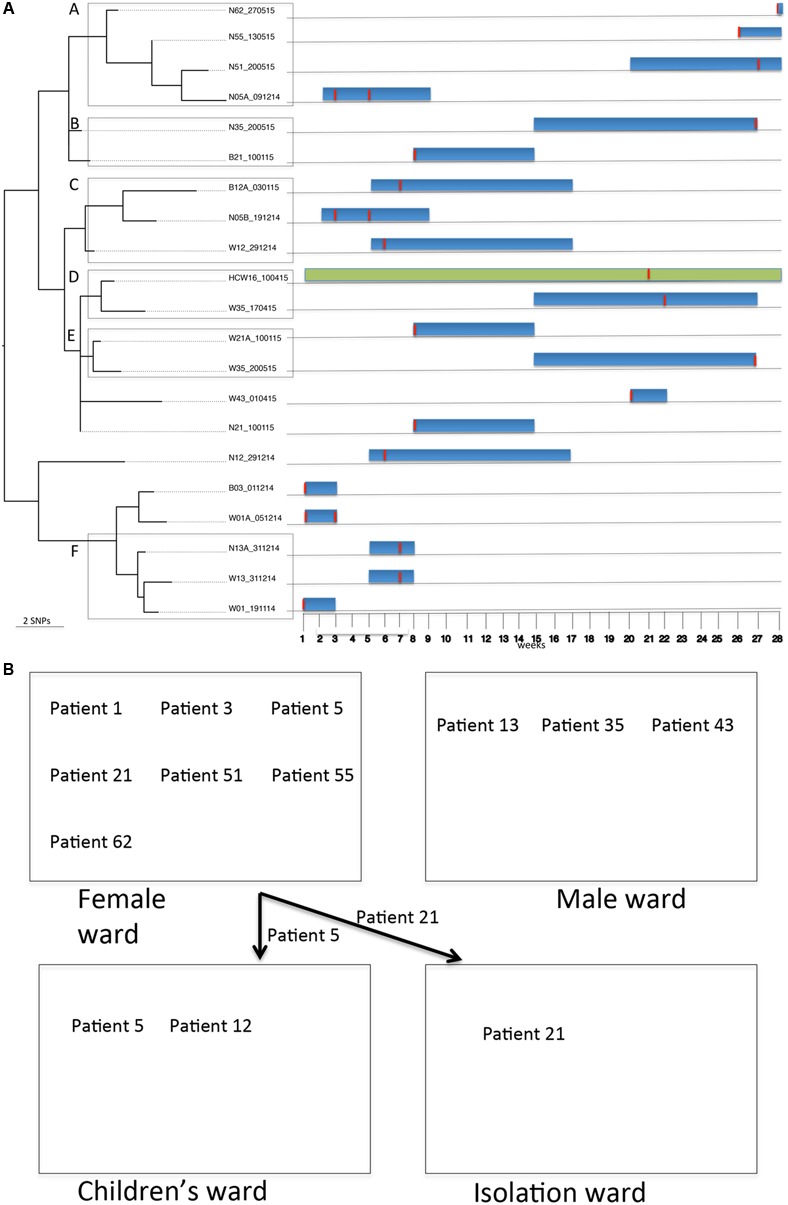
Tracing transmission events amongst burn patients and a HCW in BAPS cluster population 2 (*S. aureus* ST250). **(A)** Blue and green horizontal bars represent the length of stay (in weeks) of burn patients and HCW, respectively, in the burn unit. The red vertical lines represent the time point at which patients or HCWs tested positive for ST250-IV MRSA. **(B)** Relative positions of burn patients in the female, male, children’s and isolation wards during their stay in the burn unit.

For BAPS population 2, sequence comparisons (*S. aureus* COL reference genome) identified 1,729 core SNPs (among a core genome of 2.6 Mbp) among the 24 isolates that belonged to ST8 (*n* = 2), ST250 (*n* = 21) and ST2021 (*n* = 1). ST250 and ST2021 are single locus variants of ST8 at locus *yqi*L and *aro*E, respectively. Of note, this population was collected from two HCWs and 14 patients. Twenty-two of the 24 isolates were MRSA (ST8 and ST250). Possible transmission events were investigated in detail amongst the ST8 and ST250 subpopulation. The ST8 subpopulation identified an MRSA from a patient who was colonized on admission and an MSSA from a HCW. The ST8 MRSA lacked the PVL genes and the arginine catabolic mobile element. The SNP difference between these two isolates was 1,048 core SNPs (among a core genome of 2.6 Mbp). This SNP difference argues against a possible direct in-hospital transmission event. Further, 21 *S. aureus* isolates obtained during longitudinal sampling from 11 patients (i.e., patients 1, 3, 5, 12, 13, 21, 35, 43, 51, 55, and 62) and a HCW (HCW16) were identified as ST250. Alignment of these isolates against the reference genome of *S. aureus* COL (ST250) identified 85 core SNPs (among a core genome of 2.6 Mbp) with SNP differences between individual pairs of isolates being ≤40. Interestingly, isolates from patients who were already colonized on admission were interspersed on the ST250 phylogenetic tree (**Figure 3A**) and clustered with isolates from patients who probably acquired the strain in the burn unit, suggesting multiple introductions and spread of ST250 into the unit. Further, multiple isolates from different sites (nose, wound, and blood) and at different time points from individual patients served as a source of transmission to other patients. For example, isolates from group A (i.e., patients 5, 51, 55, and 62) differed by 23 core SNPs (among a core genome of 2.7 Mbp). All patients in group A initially resided in the adult female ward. However, patient 5 was transferred to the children’s ward after 2 weeks (**Figure 3B**). Patients 55 and 62 were already colonized on the day of admission. Interestingly, the hospital stay of patient 51 overlapped with that of patients 55 and 62, while there was no direct epidemiological link between patient 5 and any of the patients in this group. It is nevertheless likely that patient 51 acquired the ST250 strain from the patients already colonized. In the case of groups B and E, core SNP differences of six and eight were detected, respectively (among core genomes of 2.7 Mbp). The hospital stays of these two patients overlapped at the time of discharge of patient 21 and admission of patient 35. Notably, patient 21 tested positive (blood culture and wound) on admission and was treated before discharge. Patient 21 resided in the adult female ward for a week before being transferred to the isolation ward, while patient 35 resided in the male ward (**Figure 3B**). This may suggest that patient 35 obtained the strain indirectly from patient 21 (e.g., through fomites or the common dressing room used by both patients). The number of core SNP differences of isolates in group C (patients 5 and 12) was 29 (among a core genome of 2.7 Mbp) and stays of these patients overlapped in the childrens’ ward (**Figure 3B**). It is thus likely that both patients obtained the *S. aureus* strain from a common source or that patient 12 obtained the strain directly from patient 5 after being colonized from contaminated fomites in the adult female ward. In this case patient 12 acquired the strain first in the burn wound and subsequently contracted a blood stream infection. On the other hand, patient 35 was colonized with the ST250 *S. aureus* strain that was highly related to the strain previously carried by HCW16 in group D. The core SNP differences among group D isolates was 9 (among a core genome of 2.7 Mbp) suggesting that HCW16 transferred the strain to patient 35, or that both participants obtained the strain from another common source. Unfortunately, patient 35 was still colonized with the ST250 strain in the nares and wound upon discharge from the burn unit into the community (groups B and E). The hospital stay of patients in Group F [patients 1 (female ward) and 13 (male ward)] with a core SNP difference of 20 (among a core genome of 2.7 Mbp) did not overlap, suggesting that environmental contamination including air, a common dressing room or a non-screened HCW/patient may have been the source through which patient 13 acquired the ST250 strain. This explanation is plausible since patient 1 was already colonized upon admission and the patient still carried the strain when discharged from the burn unit.

**FIGURE 4:**
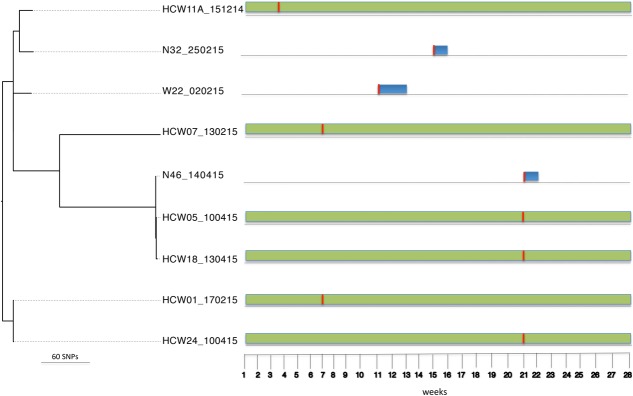
Tracing transmission events amongst burn patients in BAPS cluster population 3 (*S. aureus* ST15). Blue and green horizontal bars represent the length of stay (in weeks) of burn patients and HCWs, respectively, in the burn unit. The red vertical lines represent the time point at which patients tested positive for ST15.

Bayesian Analysis of Population Structure population 3 comprised *S. aureus* isolates from six HCWs and seven patients. These isolates belong to ST15 (*n* = 9), ST2434 (*n* = 1), ST3249 (*n* = 1), and one untypeable strain (*n* = 1). ST2434 and ST3249 are single locus variants of ST15 at the *glp*F and *pta* loci, respectively. Sequence comparison (reference genome W22_020215) among isolates in this population identified 1,426 core SNPs (among a core genome of 2.6 Mbp). The ST15 group included three patients (i.e., patients 22, 32, and 46) who were already colonized on admission and six HCWs (i.e., HCWs 1, 5, 7, 11, 18, and 24) who acquired the ST15 strain at different point in time during the study period (**Figure 4**). Amongst this group, 772 core SNP differences (among a core genome of 2.6 Mbp) were identified. The SNP difference between isolates from patient 32 and HCW 11 was 492 core SNPs (among a core genome of 2.7 Mbp) indicating that transmission events are unlikely. The SNP difference between isolates from patient 46 and HCWs 5 and 18 was 16 core SNPs (among a core genome of 2.7 Mbp). Transmission events in this case could not be inferred because patient 46 carried the ST15 isolate upon admission and the HCWs acquired the ST15 *S. aureus* strain prior to admission of patient 46. This suggests that the patient and HCWs may have acquired the strain from a common source outside the burn unit. The SNP difference between isolates from HCWs 1 and 24 was 3 core SNPs (among a core genome of 2.7 Mbp), showing that these isolates are highly related. This implies that both HCWs were colonized from a common source or a HCW-to-HCW transmission event.

For BAPS population 4, *S. aureus* isolates were obtained from 10 patients. These isolates belonged to ST1 (*n* = 1), ST5 (*n* = 2), ST6 (*n* = 1), ST3248 (*n* = 2) and ST3251 (*n* = 5). ST3248 is a single locus variant of ST1 at the *aro*E locus, ST6 is locus variant of ST5 at the *arc*C and *yqi*L loci. ST3251 is a variant of ST3248 at the *aro*E, *gmk*, *pta*, *tpi* and *yqi*L loci. Sequence comparison [reference genome isolate N315 (ST5)] among isolates in this population identified 33,375 core SNPs (among a core genome of 2.5 Mbp). *S. aureus* isolates in the STs 1, 5, 6, and 3248 originated from patients 4, 26, 31, 34, 35, and 57, who were already carriers or infected on admission. *S. aureus* isolates from five patients (i.e., patients 35, 42, 51, 54, and 62) that belonged to ST3251 had 136 core SNP differences (among a core genome of 2.6 Mbp). Here, patient 35 may have acquired the ST3251 *S. aureus* strain indirectly from the burn unit before the admission of the patients 42, 51, 54, and 62, who already carried these strains on admission. In any case, this rules out possible in hospital transmission events (data not shown).

**FIGURE 5:**
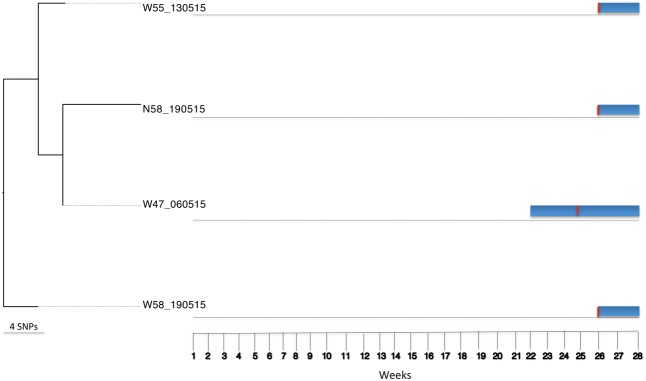
Tracing transmission events amongst burn patients in BAPS cluster population 5 (*S. aureus* ST3250). Blue horizontal bars represent the length of stay (in weeks) of burn patients in the burn unit. The red vertical lines represent the time point at which patients tested positive for ST3250.

Bayesian Analysis of Population Structure population five included six *S. aureus* isolates from four patients. These isolates belonged to ST121 (*n* = 2) and ST3250 (*n* = 4), and 1,648 core SNPs (among a core genome of 2.7 Mbp) were identified after genome comparisons (reference genome isolate W08B_161214). The ST3250 is a single locus variant of ST121 at the *yqiL* locus. Three of the four patients already carried the respective strains on admission. Among the four ST3250 isolates, two were from a patient 58 (nares and wound) and two from the wounds of patients 47 and 55 (**Figure 5**). Between these four isolates 12 core SNP differences (among a core genome of 2.7 Mbp) were found, indicating that they are highly related and most likely share a common source. The time at which patient 47 had apparently acquired the ST3250 strain preceded the time of admission of patients 55 and 58. However, since patients 55 and 58 carried their ST3250 strains already upon admission, we cannot infer direct patient-to-patient transmission events in this case (**Figure 5**).

Bayesian Analysis of Population Structure population 6 comprised five isolates from three patients and one HCW. These isolates belonged to ST45 (*n* = 2) and ST508 (*n* = 3) (data not shown). ST508 is a single locus variant of ST45 at the *aro*E locus. Sequence comparison [reference genome CA-347 (ST45)] among these isolates identified 16,001 core SNPs (among a core genome of 2.6 Mbp). Between the two ST45 isolates from patients (i.e., patients 28 and 46) there were 890 core SNP differences (among a core genome of 2.6 Mbp) and the patients’ hospital stays did not overlap. This indicates that transmission events were unlikely. On the other hand, the three ST508 isolates that originated from patient 26 and HCW21 had 17 core SNP differences (among a core genome of 2.6 Mbp). Interestingly, patient 26 was already infected with the ST508 strain on admission, while HCW21 acquired the strain around the time of admission of patient 26. This indicates a possible patient-to-HCW transmission event (data not shown).

### Detection of Antimicrobial Resistance Genes

Antibiotics consumption in the burn unit was previously shown to be high and *S. aureus* isolates were resistant to some prescribed antibiotics including gentamicin, ciprofloxacin and erythromycin (Amissah et al., 2017). Therefore, we assessed genotypic antibiotic resistances from the *de novo* assembled genomes of the 66 study isolates. The results are shown in **Figure 1**. All isolates that belonged to ST250 carried the *aac*(6′)-*aph*(2″) gene that encodes resistance to aminoglycosides (gentamicin, kanamycin, and tobramycin). One isolate in the ST8 group contained the *ant*(6)-*Ia* and *aph*(3″)-III genes conferring resistance to the aminoglycoside kanamycin. Further, the ST250 isolates carried the *blaZ* and *mecA* genes, the *cat*(pC221), and the *tetK* and *tetM* genes, which confer resistance to β-lactams (penicillin [100%] and oxacillin [33%]), chloramphenicol (50%) and tetracycline (59%) (**Figure 1**) respectively. A point mutation in the *rpoB* gene, encoding a histidine to asparagine conversion at amino acid position 481 that confers resistance to rifampicin (Aubry-Damon et al., 1998), was detected in one isolate belonging to ST5. In one isolate that belonged to ST8 the *mph*(*C*) and *msr*(*A*) genes were found, which mediate resistance to macrolides (erythromycin and clindamycin). Lastly, the *dfrG* gene encoding trimethoprim resistance, and the biocide resistance gene *norA* were detected in 21 and 100% of the sequenced isolates, respectively.

## Discussion

This study describes possible *S. aureus* transmission events in the burn unit of a tertiary care center in Ghana. MLST analysis of 66 isolates identified 16 STs including four new genotypes, with ST250 being the predominant genotype. Unsupervised Bayesian clustering grouped the isolates into six distinct populations, four of which were comprised of isolates from patients and HCWs. Most patients carried *S. aureus* on admission, and transmission events were confirmed in 25% of the cases in the burn unit.

The overall infected or carriage rate (81%) of patients with *S. aureus* on admission was high compared to 35% reported in other studies (Kooistra-Smid et al., 2004; Kaiser et al., 2011). This may have affected wound healing, as it has been shown that patients carrying *S. aureus* in the nares or throat on admission, are often eventually colonized with the same genotype in the burn wound (Kooistra-Smid et al., 2004).

The ST152 PVL-positive community-acquired methicillin susceptible *S. aureus* (CA-MSSA) is significantly predominant in Africa (Ruimy et al., 2008; Shittu et al., 2012; Egyir et al., 2014; Conceição et al., 2015; Kraef et al., 2015; Eibach et al., 2017) and has been detected in skin lesions of Buruli ulcer patients (Amissah et al., 2015; Kpeli et al., 2016). Consistent with these previous data, seven of the eight ST152 MSSA study isolates were PVL-positive (Amissah et al., 2017). In contrast, a few cases of ST152 PVL-positive community-acquired MRSA (CA-MRSA), responsible for skin and soft infections, were reported in Europe (Jappe et al., 2008; Katopodis et al., 2010; Krziwanek et al., 2011; Brauner et al., 2013) and in Haiti (Rosenthal et al., 2014). Since the PVL virulence factor is associated with necrotic skin lesion (Novick et al., 1993; Novick, 2003), carriage of ST152 PVL-positive MSSA in the wounds of burn patients may have health implications, such as delayed wound healing and could lead to invasive infection. In the present study, we hypothesize that HCWs may have served as a source for *S. aureus* ST152 transmission to patients.

The ST250 clone was the first reported cause of epidemic MRSA disease, and the dominant MRSA sequence type in the mid-1960s in Europe (Denmark, Germany, Switzerland, and United Kingdom), Uganda and Australia, and during the 1970s–1980s in Ireland (Enright et al., 2002; Shore et al., 2005; Lancashire et al., 2013). While there are currently no reports of MRSA with ST250 in these regions (Enright et al., 2002), this MRSA type has recently emerged in skin and soft tissue infections in a health care center in Ghana (Egyir et al., 2014). Further, our previous data (Amissah et al., 2017) showed a wider distribution of ST250-MRSA with the SCC*mec* IV variant (i.e., ST250-IV MRSA) from other referral health care centers in Ghana, suggesting the rapid dissemination of this strain. Our present study provides clear evidence for ST250-IV MRSA transmission events that had been suspected based on *spa*- and MLST-typing and antibiotic resistance profiles (Amissah et al., 2017). In addition, possible patient-to-patient and HCW-to-patient transmission routes could now be described based on the use of WGS. With the high number of ST250-IV MRSA-positive patients observed on admission, it seems likely that MRSA outbreaks can occur unnoticed in the investigated burn unit. In turn, this may result in prolonged patient stays, increased cost of treatment and even increased mortality rates. It is important to note that, unfortunately, infection control measures were not applied to most of the MRSA-positive patients. Only one of the 11 patients was isolated and ST250-IV MRSA-positive patients (patients 1 and 35) were dismissed from the burn unit still being positive. In addition, MRSA transmission events occurred probably due to the transfer of the ST250-IV MRSA-carrying patient 5 from one ward to another ward. Retrospectively, it must be concluded that implementation of barrier precautions, such as patient isolation, would have been an appropriate approach to limit the unwanted transmission of MRSA with ST250 in the investigated burn unit. Of note, basic hand hygiene compliance by HCWs was observed, but their knowledge on infection prevention and control measures may have been limited. Also, important resources, such as hand hygiene stations, disinfecting hand rub and disposable towels were not available in the burn unit. This is in line with previous studies, which reported that hand hygiene compliance by healthcare personnel in Ghana is generally low (Owusu-Ofori et al., 2010; Yawson and Hesse, 2013).

Analyses of the ST15 and ST508 isolates identified possible HCW-to-HCW and patient-to-HCW transmission events that further emphases lapses in infection control measures. Notably, these isolates belong to major MSSA clones that have been detected in community and healthcare settings in humans and livestock in Africa (Ghebremedhin et al., 2009; Conceição et al., 2014a,b, 2015; Egyir et al., 2014; Schaumburg et al., 2015).

Despite the low core SNP differences among *S. aureus* ST3250 isolates from the three patients who carried them, we were unable to pinpoint possible patient-to-patient transmission events for these isolates. Hence alternative explanations for the identification of the highly similar ST3250 isolates in the three patients could be that transmission may have occurred through visitors or contaminated fomites.

The spread of antibiotic resistant *S. aureus*, including MRSA, among burn patients is particularly worrisome and is an issue of major public health importance^5^. The high rates of antibiotic resistance nowadays observed in bacterial pathogens have, amongst others, been reported to correlate with the high use of antibiotics in healthcare institutions (Westh et al., 2004; Borg et al., 2010; Nakamura et al., 2012; Kim et al., 2013). Recently, we observed a high consumption of antibiotics by patients for empirical treatment in the investigated Ghanaian burn unit (Amissah et al., 2017). This is an unwanted situation, and administering appropriate antibiotics at the right dosage will be helpful in preventing the emergence of highly resistant strains. Particularly in African countries like Ghana, where antibiotics are readily accessible in pharmacies, the emergence of antibiotic resistant *S. aureus* amongst burn patients may have been the consequence of this high consumption of antibiotics in the community.

While clear evidence for *S. aureus* transmission has been obtained, our study has nevertheless some limitations. MRSA carriage or infection upon admission was previously reported to be associated with prior hospitalization with a length of stay of ≥7 days and surgery within the past 6 months (Wibbenmeyer et al., 2010). Although referrals of patients were often made from the Korle Bu Teaching Hospital’s ICU, our study lacked patient data prior to their referral to the burn unit. Such patient data would have been informative in determining the risk factors that predisposed patients to MRSA carriage or infection already on admission. Secondly, the routes of acquisitions were elucidated for only two of the eight nosocomial transmission events observed in patients. In this study, we were unable to identify other possible sources of MRSA transmission, such as contaminated fomites or visitors, which could perhaps have explained the indirect *S. aureus* acquisition by patients whose hospital stays did not overlap.

## Conclusion

Our present investigations have unveiled the introduction of CA-MSSA including PVL-positive CA-MSSA and hospital-acquired MRSA previously dominant in Europe, as well as nosocomial *S. aureus* transmission events among patients and HCWs, in the burn unit of a tertiary health care center in Ghana. WGS thus demonstrates the importance of infection control measures and appropriate antibiotic stewardship. Clearly, the implementation of relatively cheap typing methods, such as multiple-locus variable number tandem repeat fingerprinting and *spa*-typing, or antibiotic susceptibility testing, allow the monitoring of suspected MRSA outbreaks in health care centers of resource-limited countries like Ghana. Nonetheless, unambiguous identification of MRSA transmission events and outbreaks will require the more sophisticated WGS-based comparison of individual isolates.

## Author Contributions

NA, AA, TvdW, JvD, JR, and YS conceived and designed the experiments. NA, LvD, CT, and IP performed the experiments. NA, AB, SB, DB, and TPS analyzed the data. AA, AO-W, TvdW, AF, TS, JvD, YS, TPS, and JR contributed reagents/materials/analysis tools. NA, AB, JvD, YS, TPS, and JR wrote the paper.

## Conflict of Interest Statement

The authors declare that the research was conducted in the absence of any commercial or financial relationships that could be construed as a potential conflict of interest.

## Footnotes

1 https://github.com/tseemann/snippy

2 https://github.com/tseemann/mlst

3 https://github.com/tseemann/abricate

4 http://tree.bio.ed.ac.uk/software/figtree/

5 https://amr-review.org/
